# Supplementary material for: The neuronal calcium sensor NCS-1 regulates the phosphorylation state and activity of the Gα chaperone and GEF Ric-8A
Source: eLife. 2023 Nov 29;12:e86151. doi: 10.7554/eLife.86151 (PMC10732572; doi:10.7554/eLife.86151)
Supplement: Figure 2—source data 1. [file elife-86151-fig2-data1.zip › SourceData-Fig2/Figure2A-gel.pptx]

## Slide 1
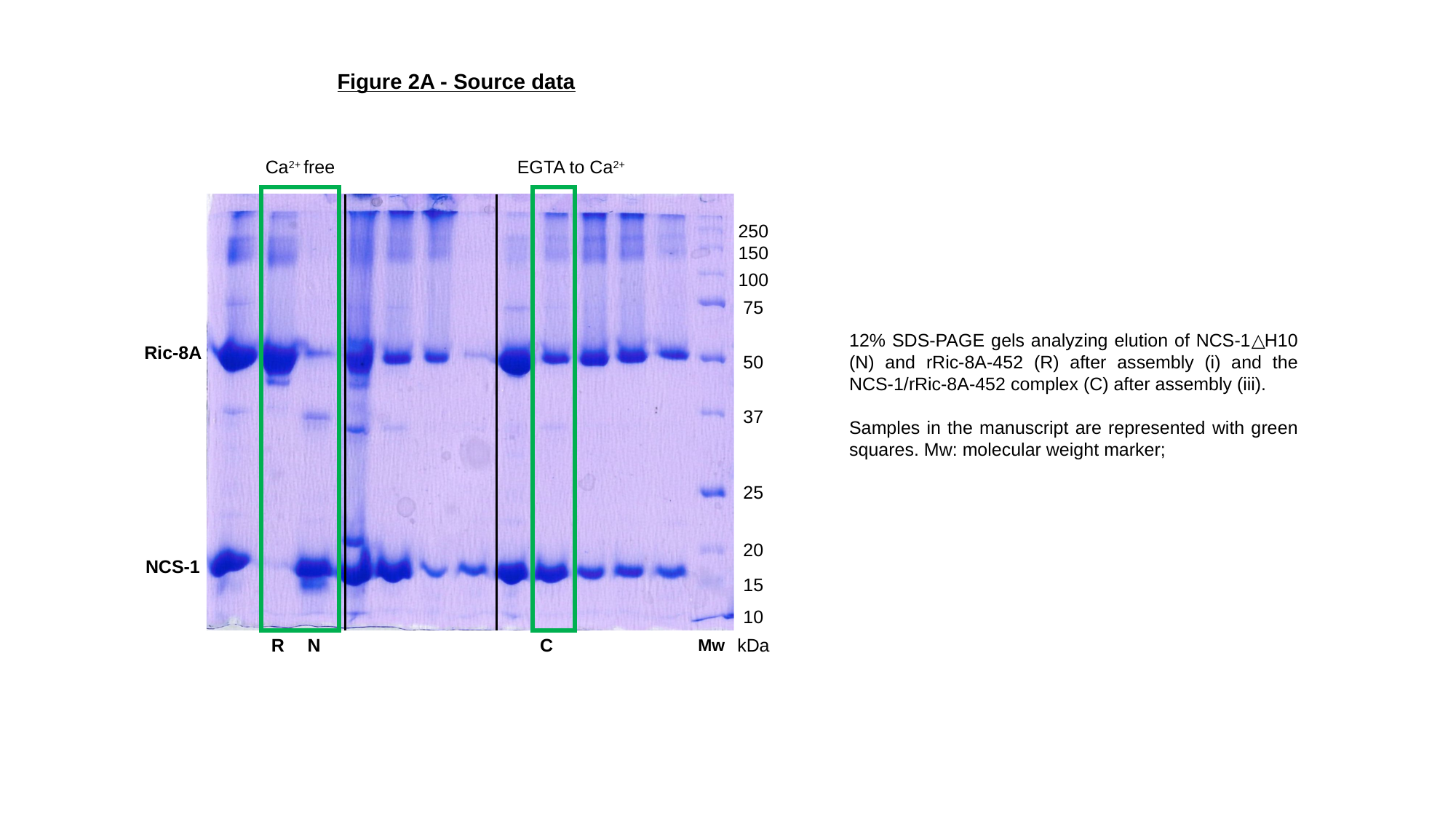

Figure 2A - Source data
Ca2+ free
EGTA to Ca2+
250
150
100
75
12% SDS-PAGE gels analyzing elution of NCS-1△H10 (N) and rRic-8A-452 (R) after assembly (i) and the NCS-1/rRic-8A-452 complex (C) after assembly (iii).
Samples in the manuscript are represented with green squares. Mw: molecular weight marker;
Ric-8A
50
37
25
20
NCS-1
15
10
R
N
C
kDa
Mw
