# Supplementary figures and images for: The neuronal calcium sensor NCS-1 regulates the phosphorylation state and activity of the Gα chaperone and GEF Ric-8A
Source: eLife. 2023 Nov 29;12:e86151. doi: 10.7554/eLife.86151 (PMC10732572; doi:10.7554/eLife.86151)

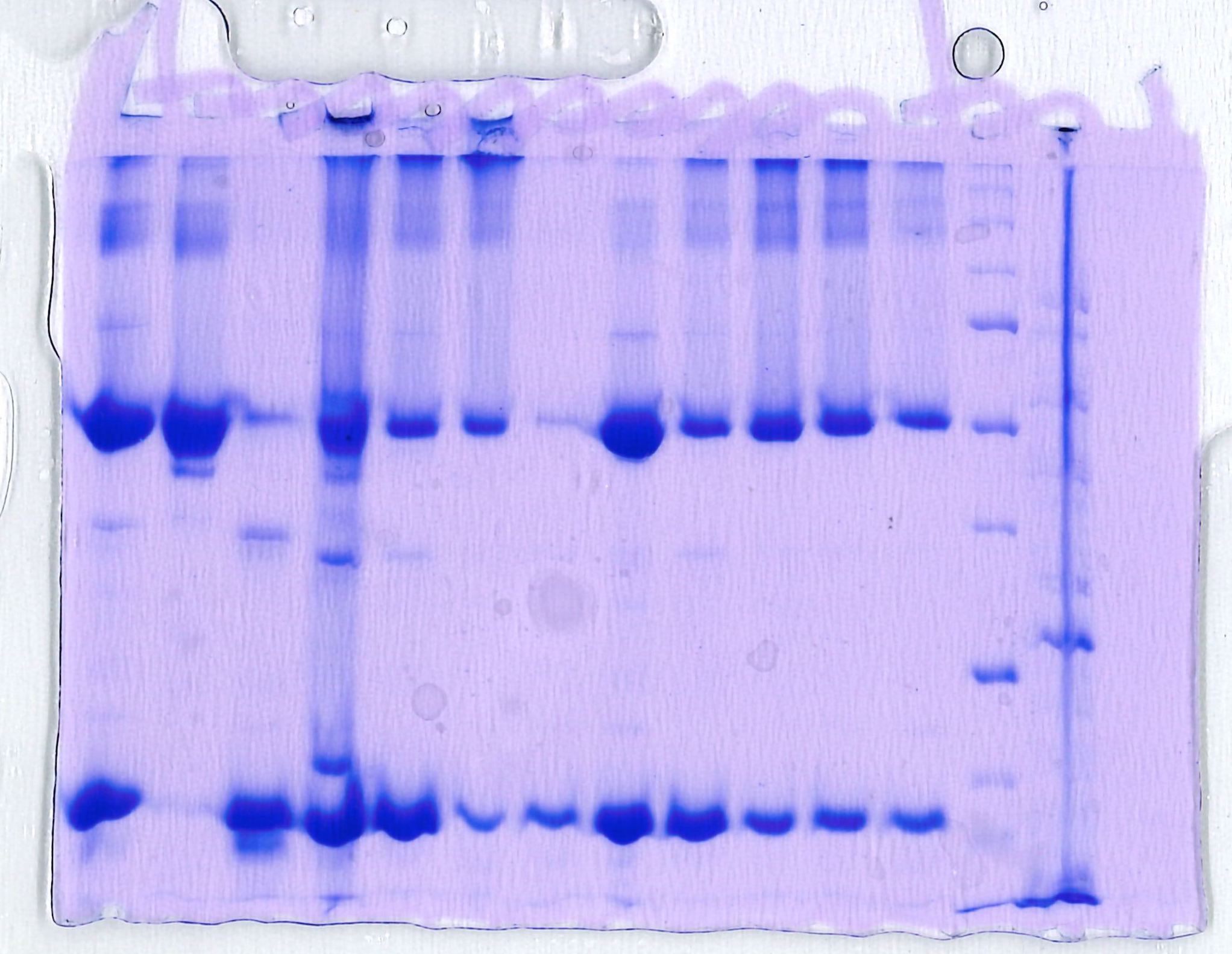

Supplement: Figure 2—source data 1. [file elife-86151-fig2-data1.zip › SourceData-Fig2/Figure2A-gel-unedited.jpg]

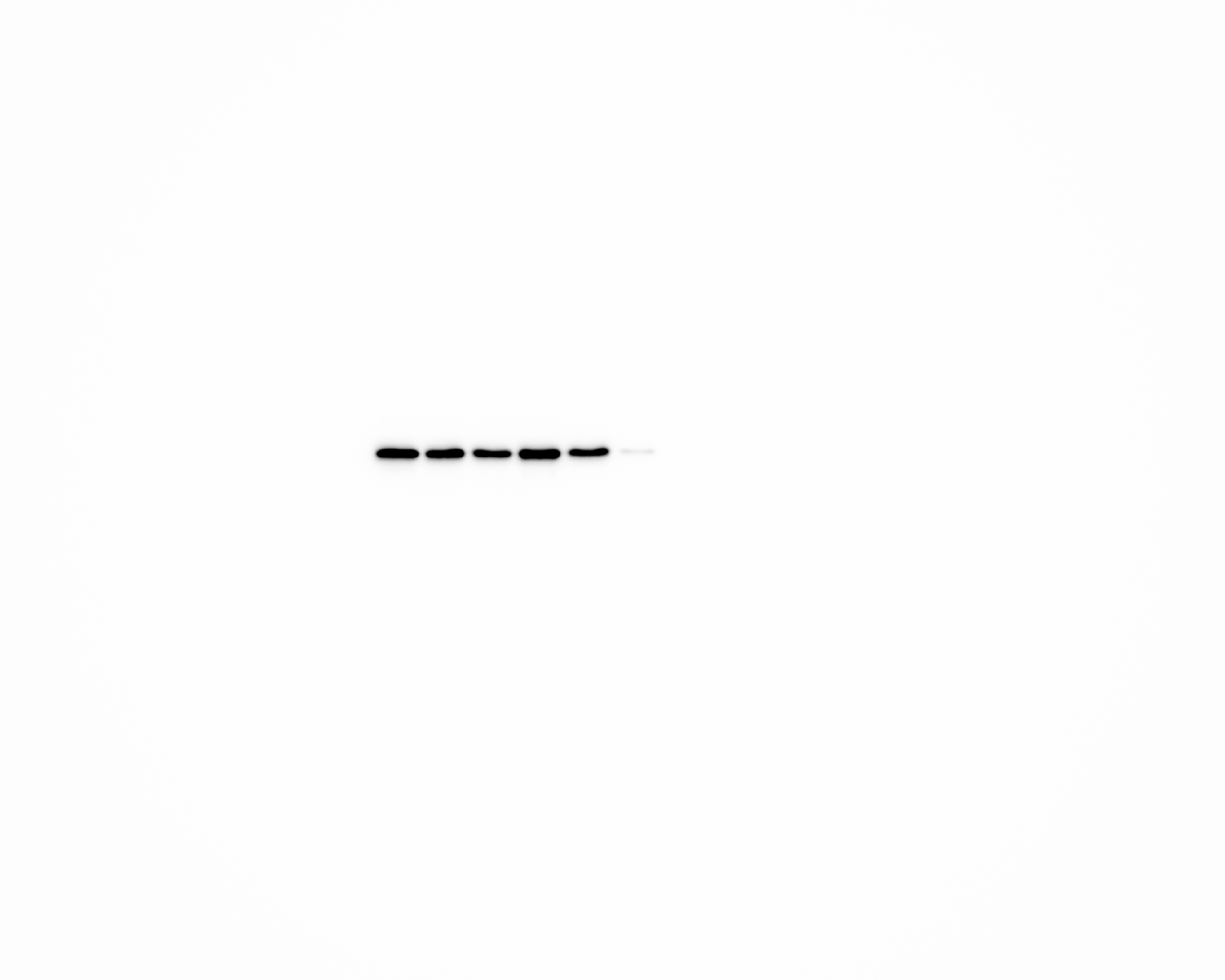

Supplement: Figure 2—source data 1. [file elife-86151-fig2-data1.zip › SourceData-Fig2/Figure 2C INPUT NCS1.tif]

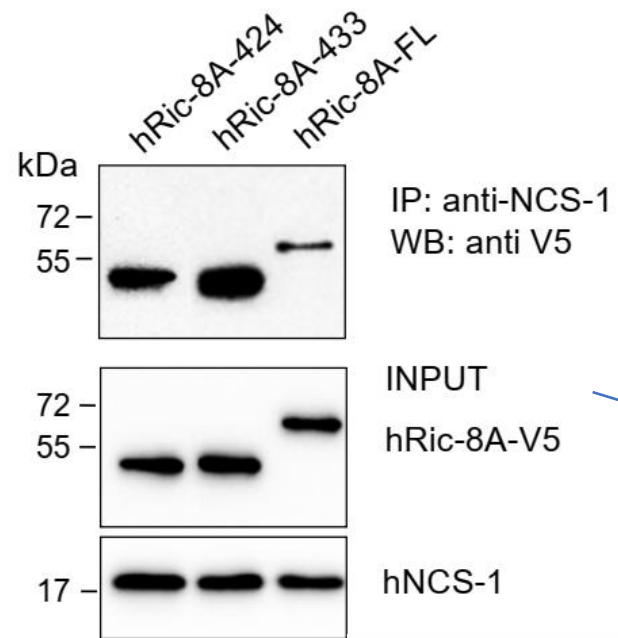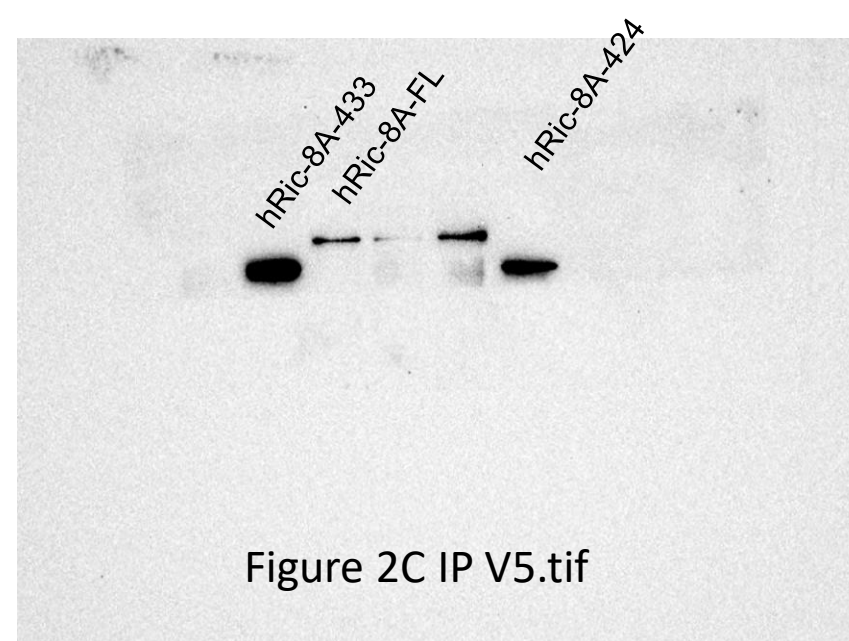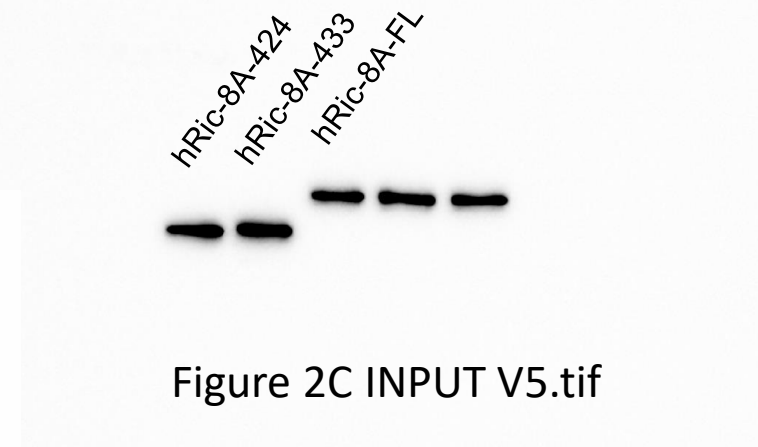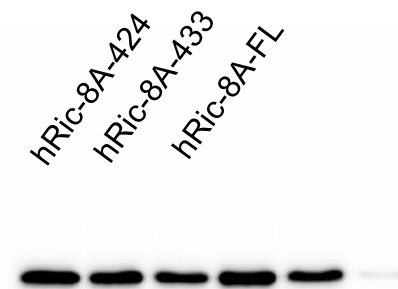

Supplement: Figure 2—source data 1. [file elife-86151-fig2-data1.zip › SourceData-Fig2/Figure 2C raw data legend.pdf]

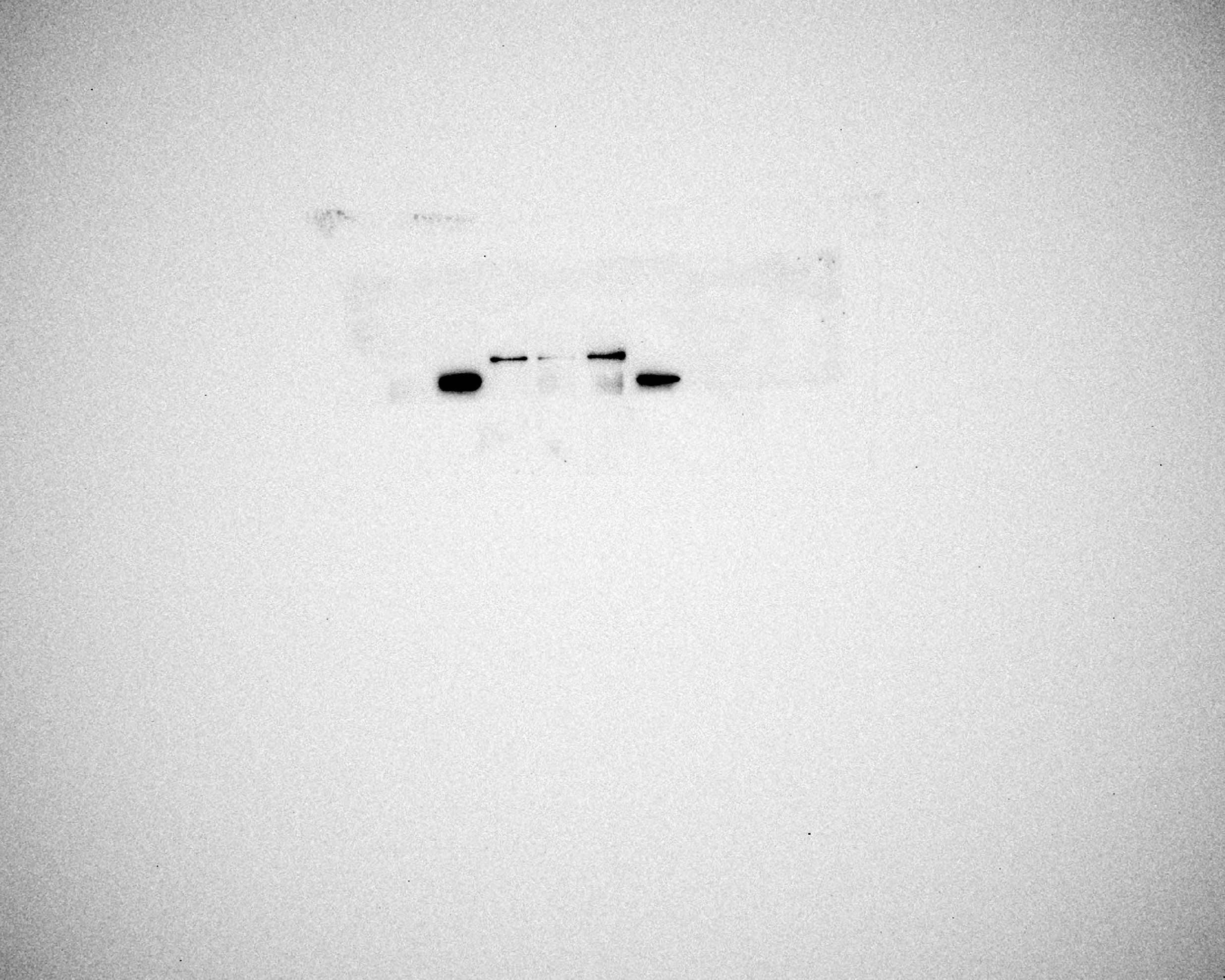

Supplement: Figure 2—source data 1. [file elife-86151-fig2-data1.zip › SourceData-Fig2/Figure 2C IP V5.tif]

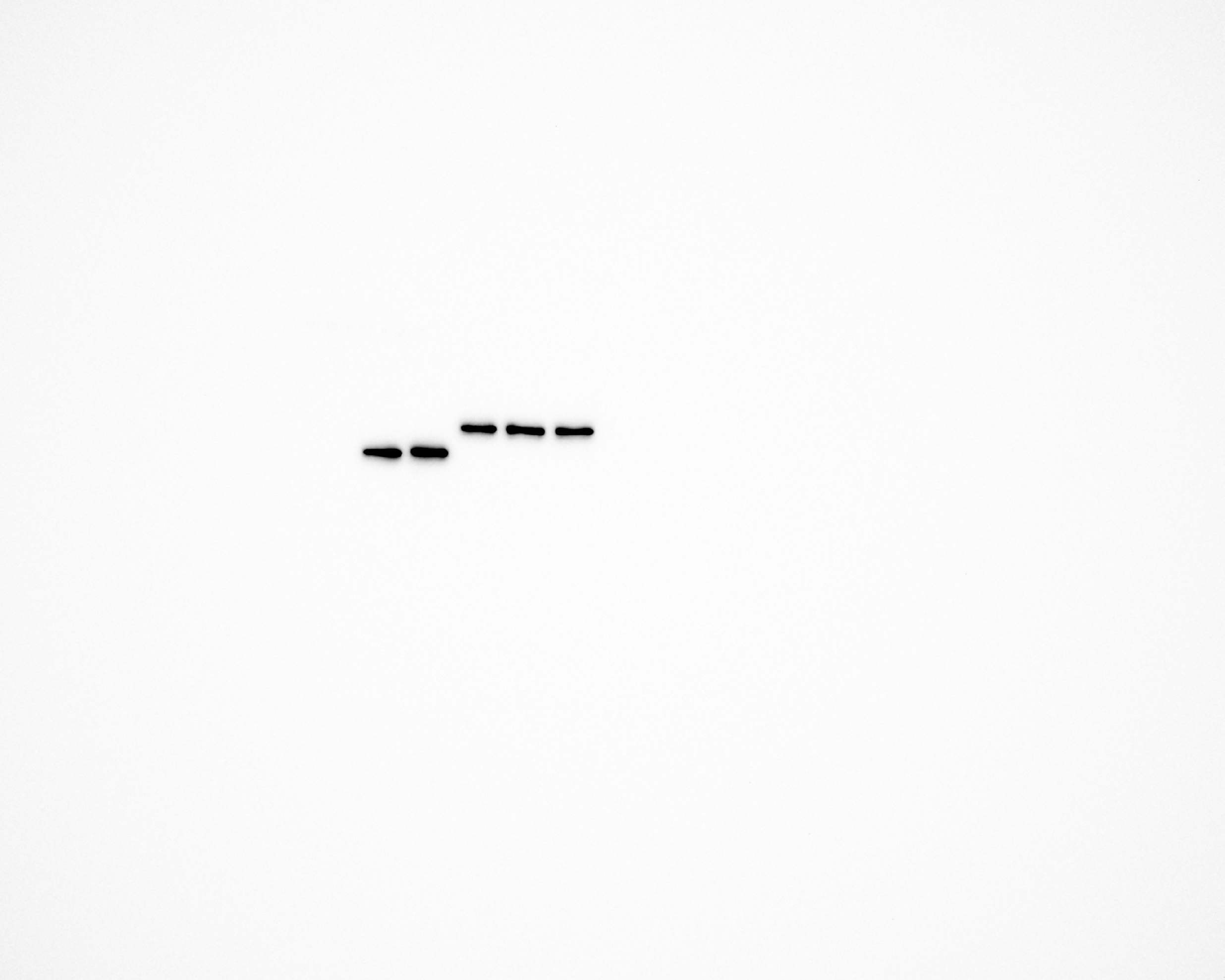

Supplement: Figure 2—source data 1. [file elife-86151-fig2-data1.zip › SourceData-Fig2/Figure 2C INPUT V5.tif]

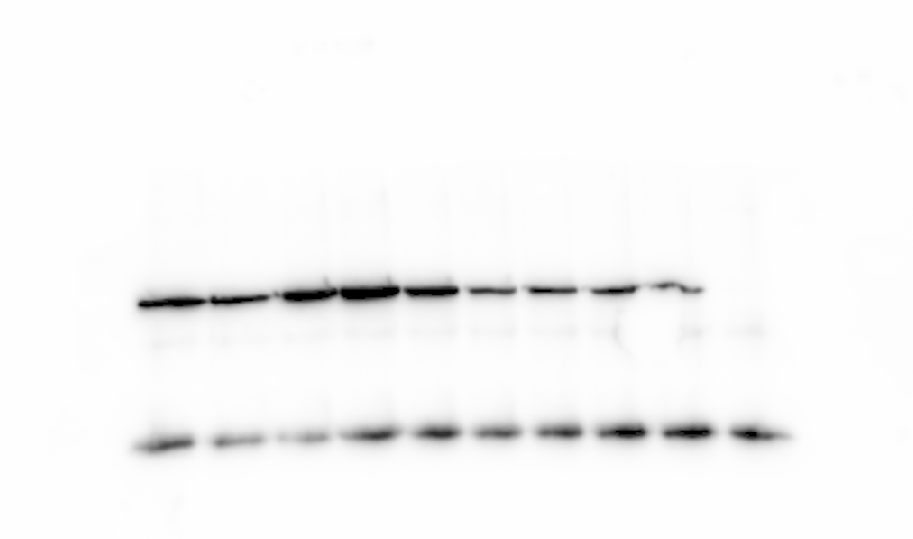

Supplement: Figure 3—source data 1. [file elife-86151-fig3-data1.zip › SourceData-Figure3D/Gel3 IP.tiff]

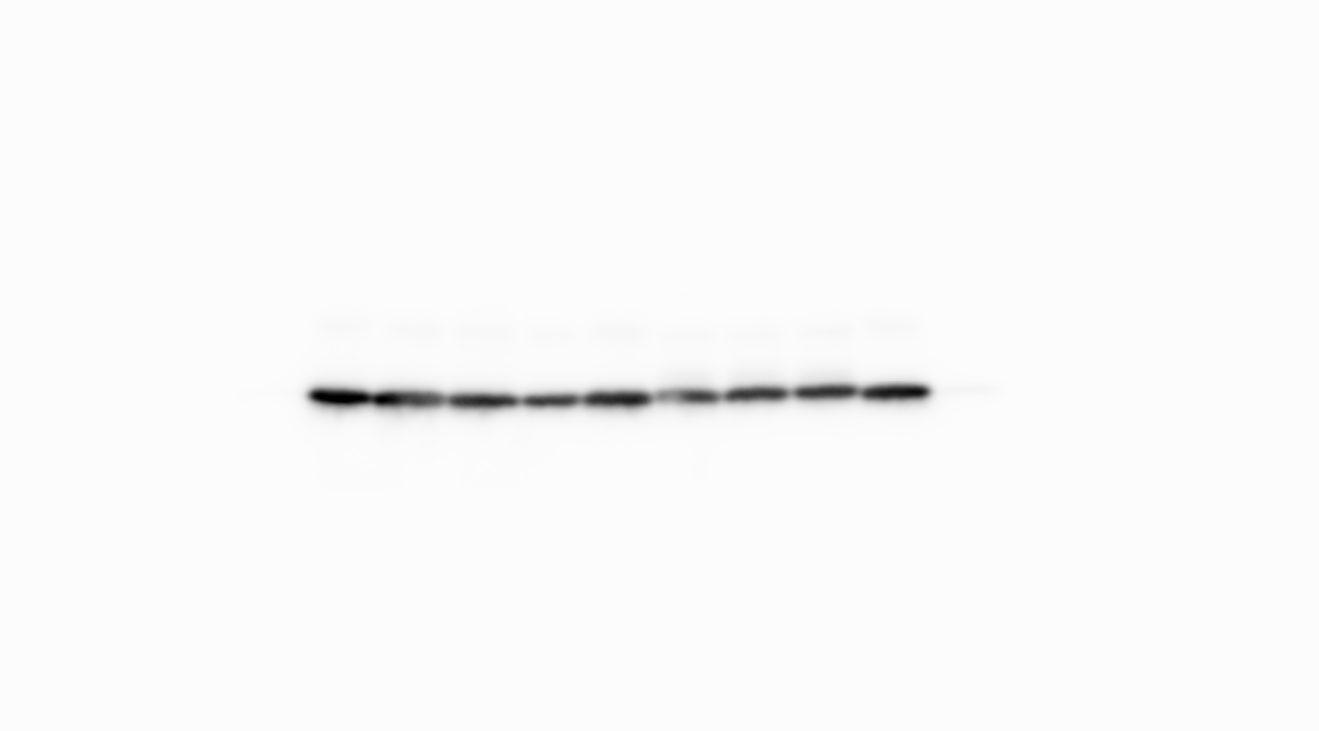

Supplement: Figure 3—source data 1. [file elife-86151-fig3-data1.zip › SourceData-Figure3D/Gel3 Input NCS1.tif]

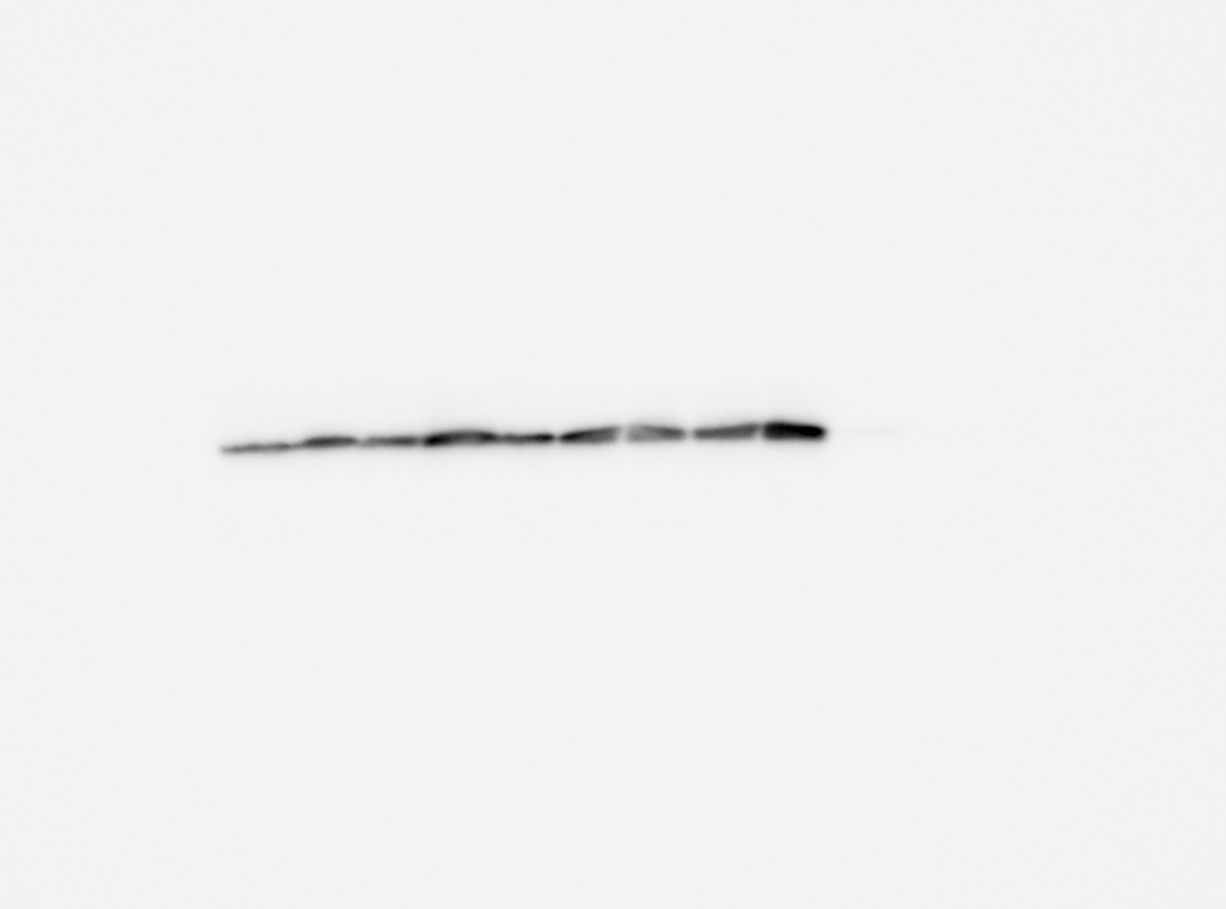

Supplement: Figure 3—source data 1. [file elife-86151-fig3-data1.zip › SourceData-Figure3D/Gel2 Input NCS1.tif]

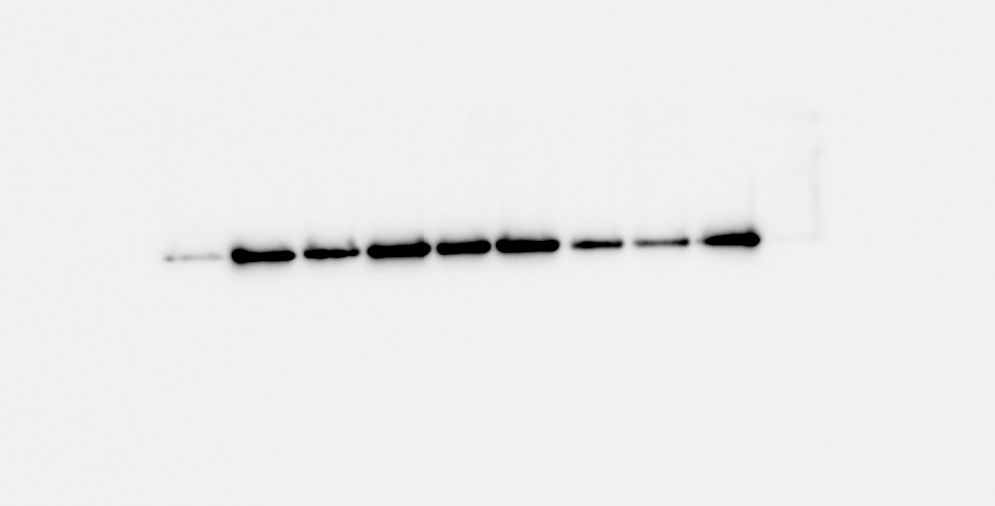

Supplement: Figure 3—source data 1. [file elife-86151-fig3-data1.zip › SourceData-Figure3D/Gel2 IP.tiff]

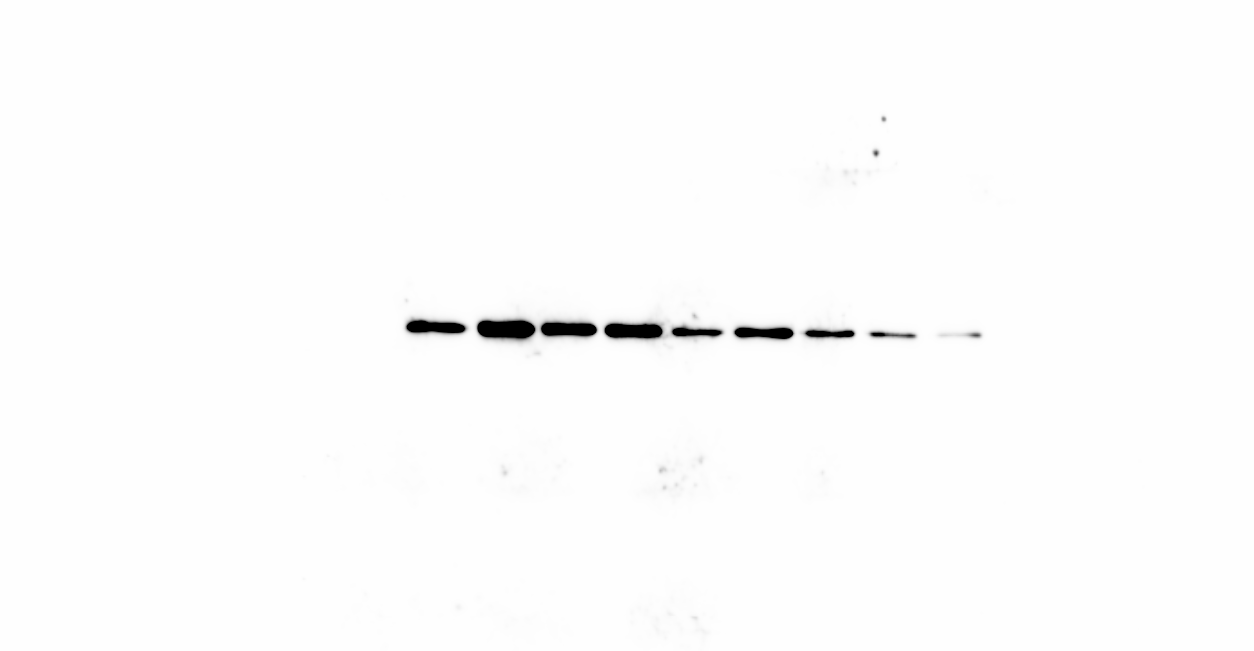

Supplement: Figure 3—source data 1. [file elife-86151-fig3-data1.zip › SourceData-Figure3D/Gel1 IP.tiff]

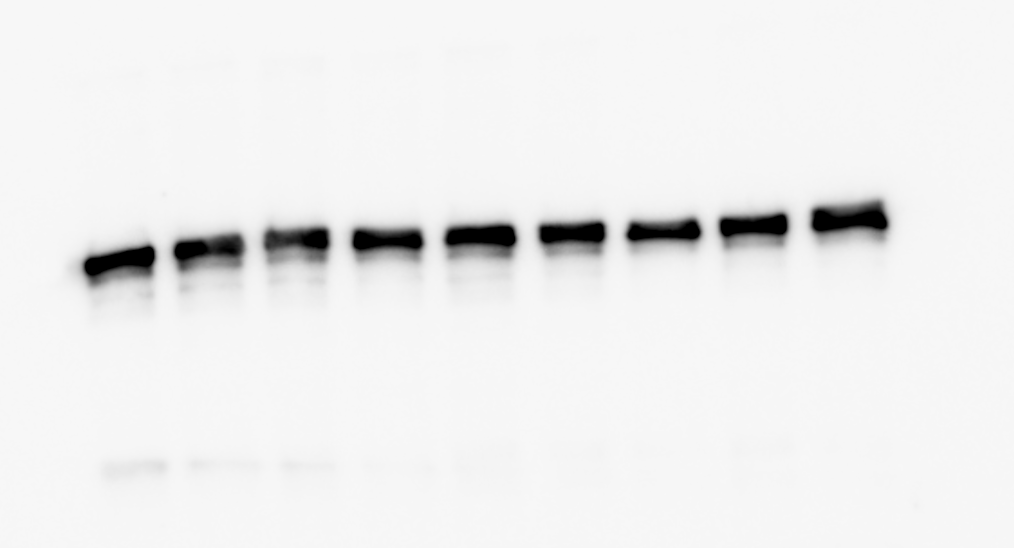

Supplement: Figure 3—source data 1. [file elife-86151-fig3-data1.zip › SourceData-Figure3D/Gel1 Input NCS1.tif]

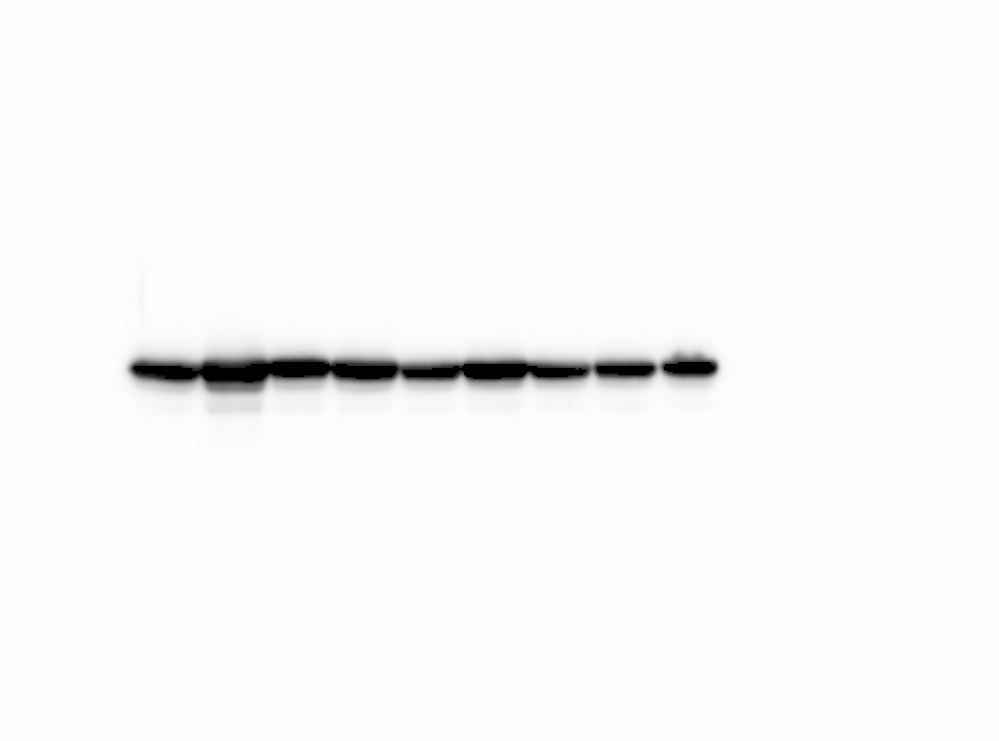

Supplement: Figure 3—source data 1. [file elife-86151-fig3-data1.zip › SourceData-Figure3D/Gel1 Input Ric8V5.tif]

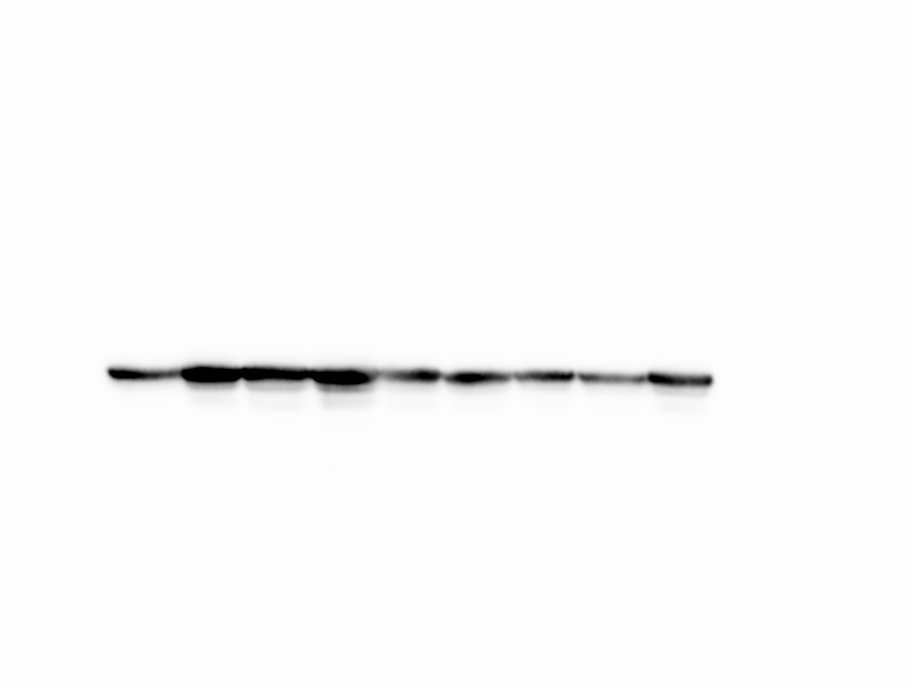

Supplement: Figure 3—source data 1. [file elife-86151-fig3-data1.zip › SourceData-Figure3D/Gel2 Input Ric8V5.tif]

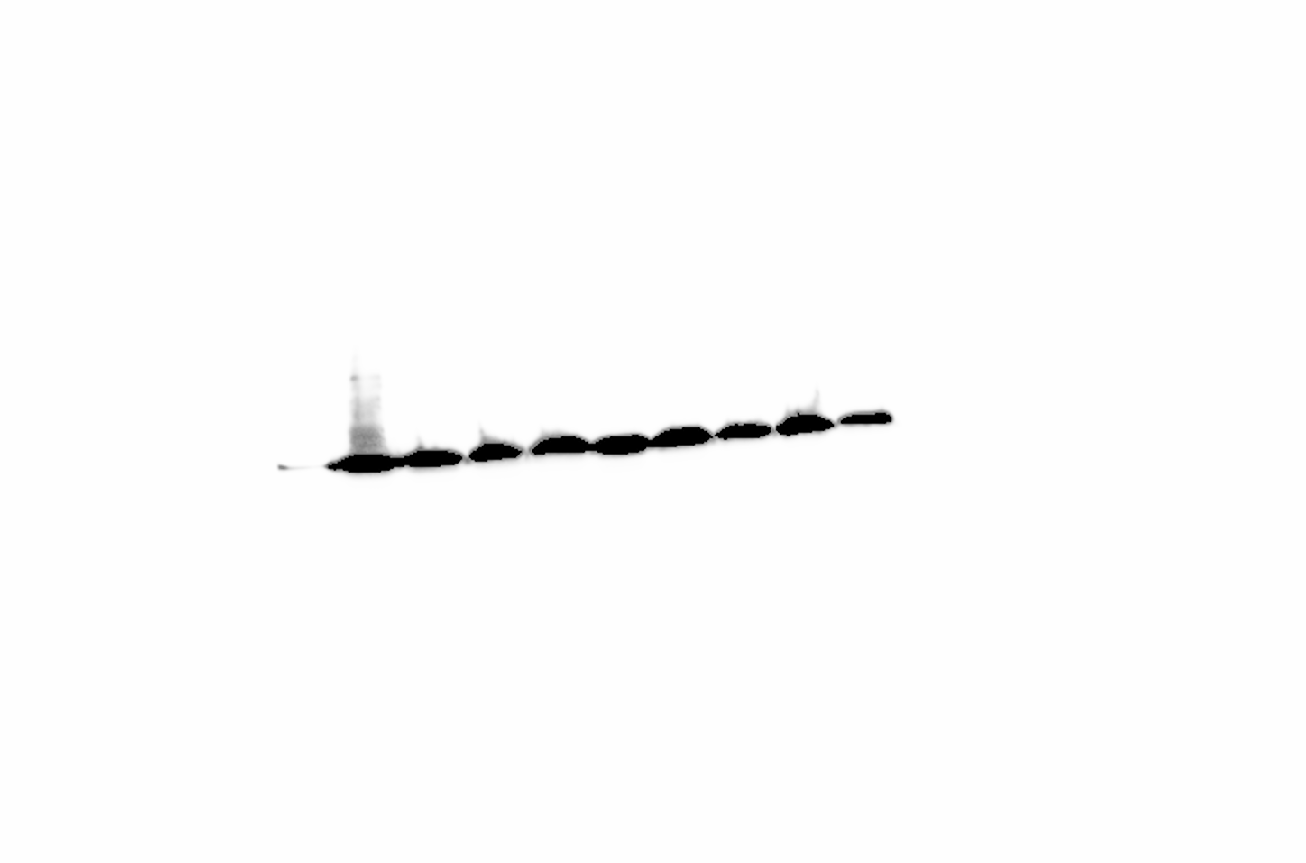

Supplement: Figure 3—source data 1. [file elife-86151-fig3-data1.zip › SourceData-Figure3D/Gel3 Input Ric8V5.tif]

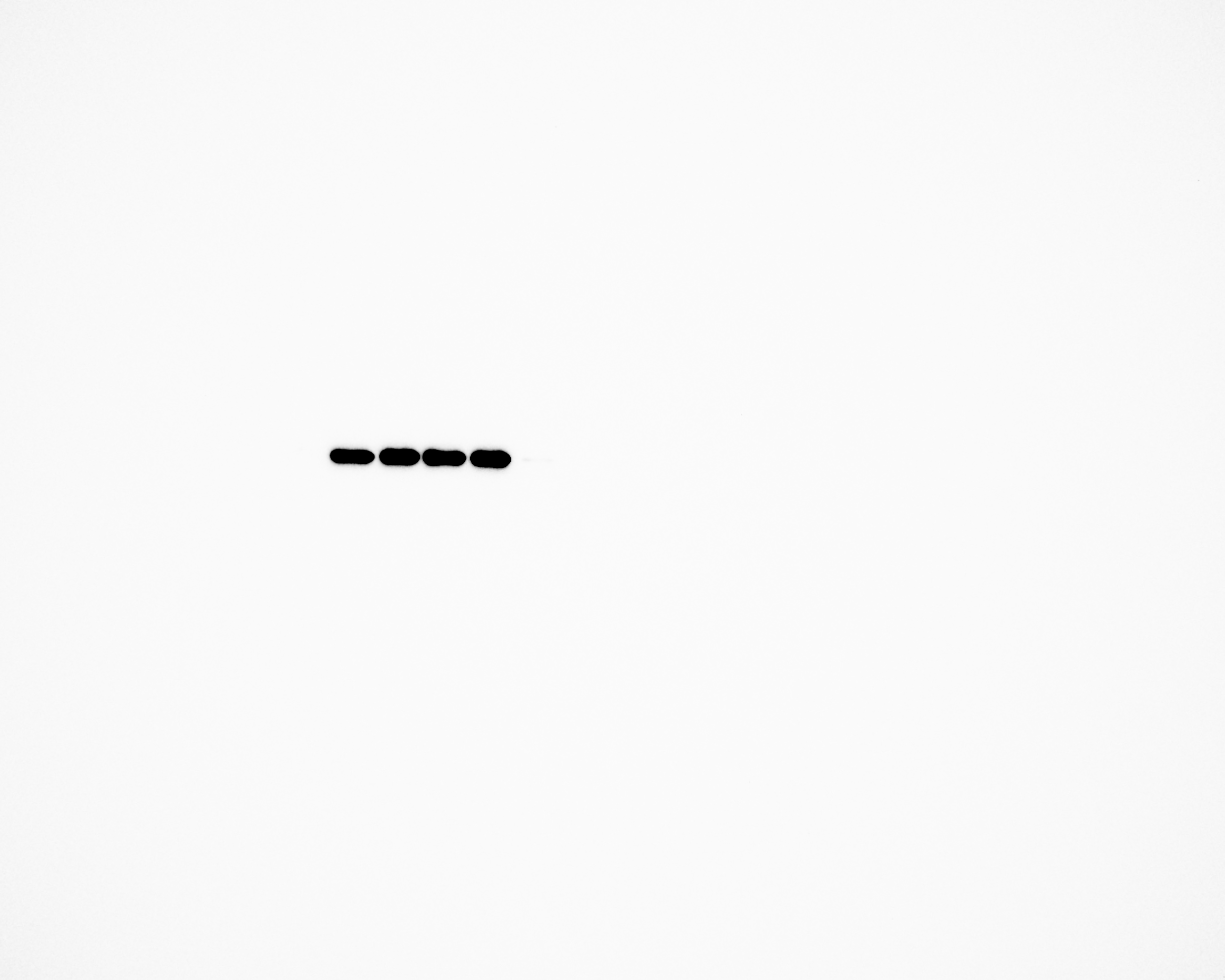

Supplement: Figure 5—source data 1. [file elife-86151-fig5-data1.zip › SourceData-Fig6/Figure 6A INPUTV5.tif]

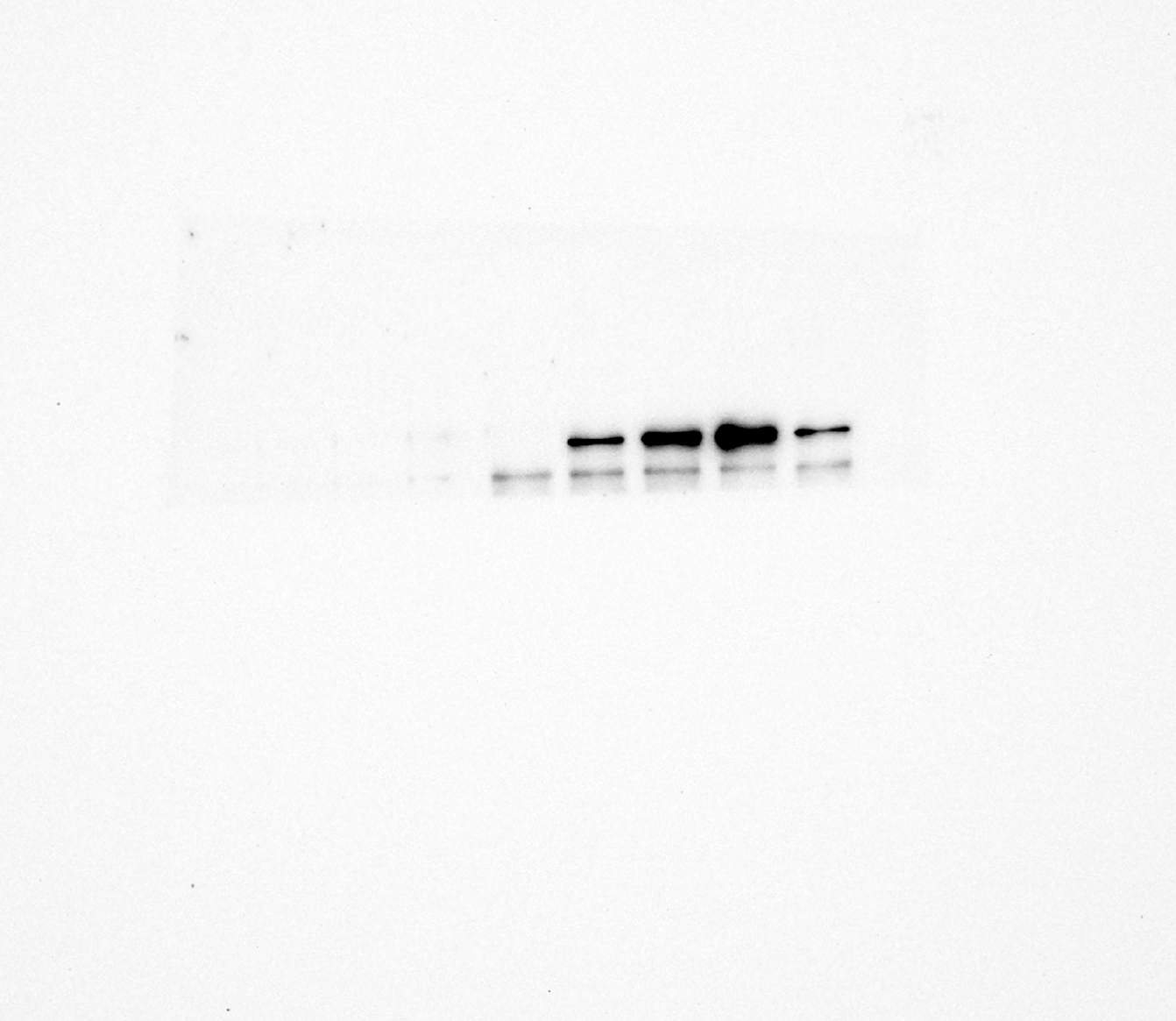

Supplement: Figure 5—source data 1. [file elife-86151-fig5-data1.zip › SourceData-Fig6/Figure 6A IP V5.tif]

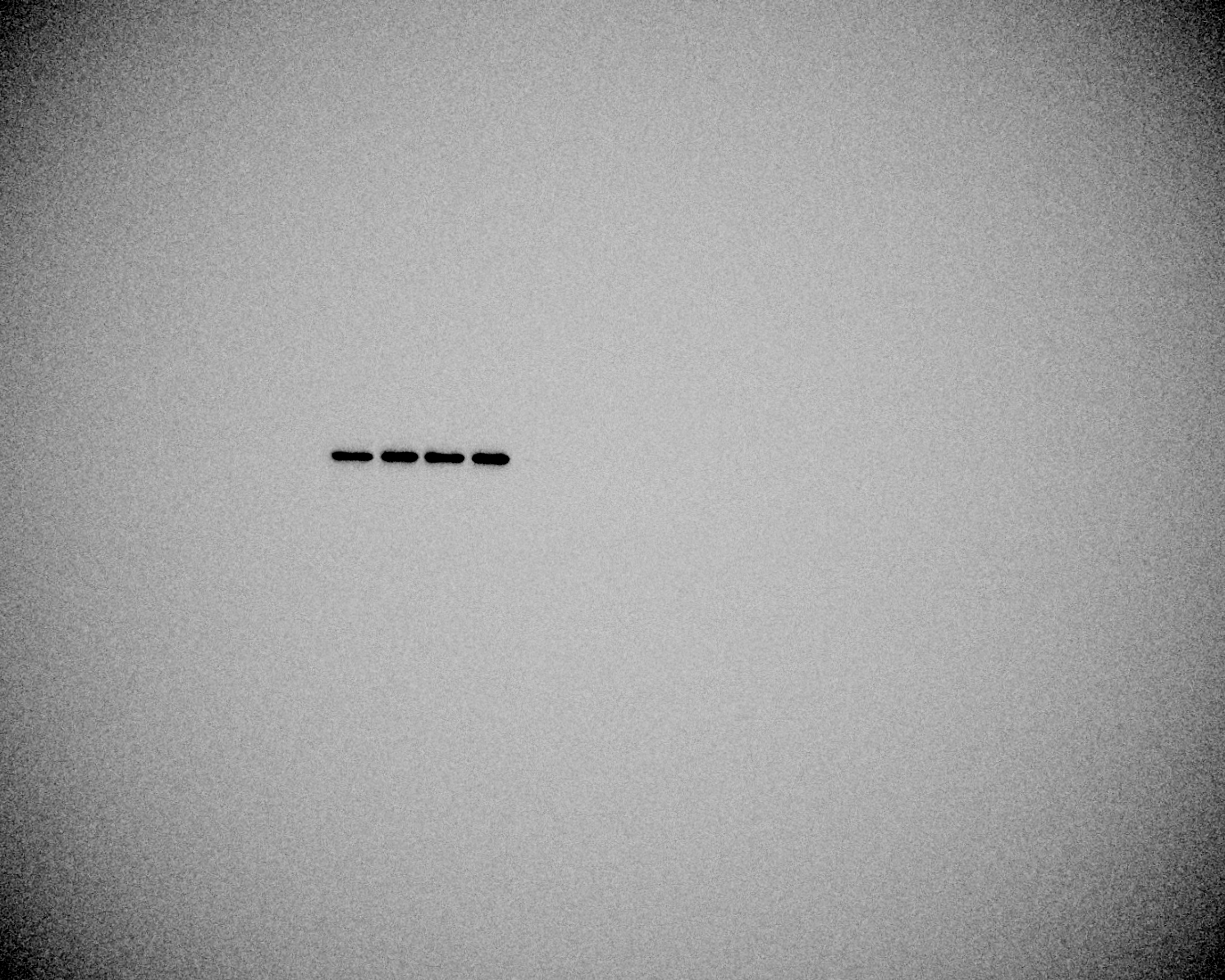

Supplement: Figure 5—source data 1. [file elife-86151-fig5-data1.zip › SourceData-Fig6/Figure 6A INPUT NCS1.tif]
