# Supplementary material for: The neuronal calcium sensor NCS-1 regulates the phosphorylation state and activity of the Gα chaperone and GEF Ric-8A
Source: eLife. 2023 Nov 29;12:e86151. doi: 10.7554/eLife.86151 (PMC10732572; doi:10.7554/eLife.86151)
Supplement: Figure 3—source data 1. [file elife-86151-fig3-data1.zip › SourceData-Figure3D/Figure 3D raw data legend.pdf]

GEL1

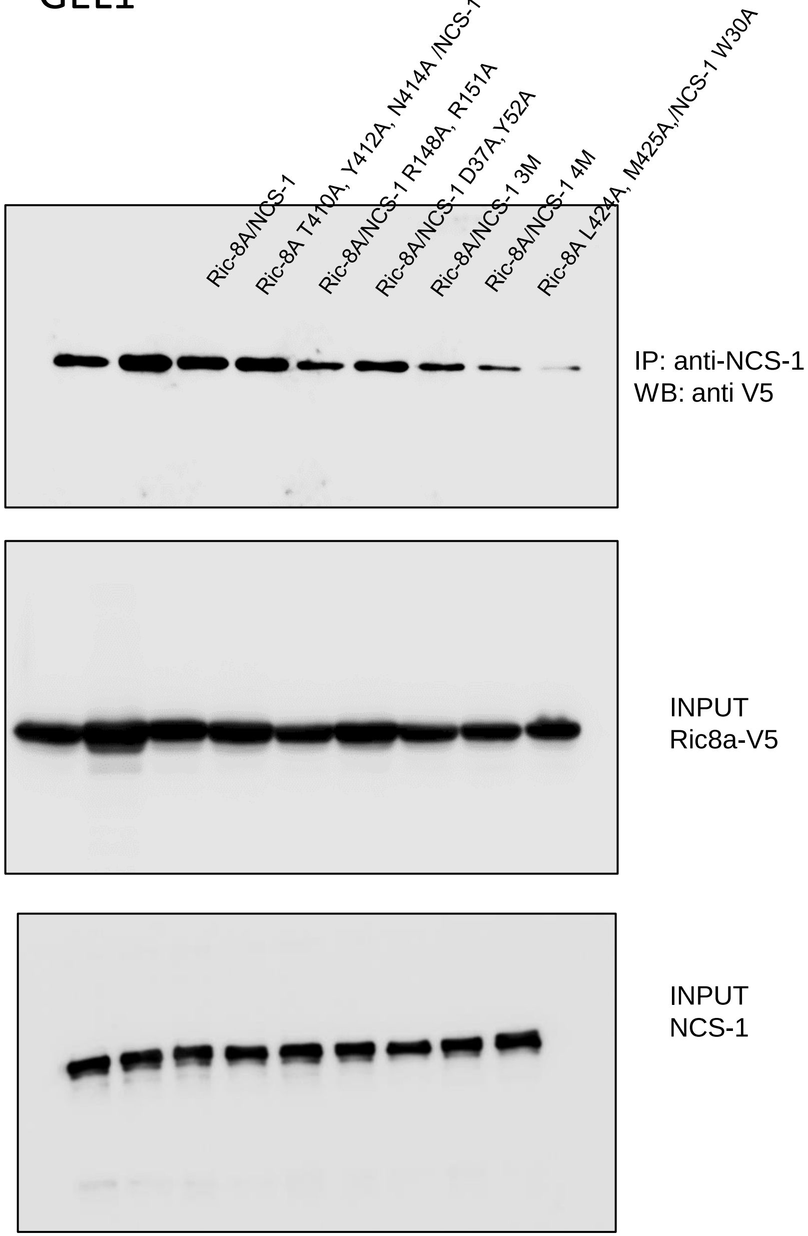
Ric-8A/NCS-1
Ric-8A T410A, Y412A, N414A /NCS-1
Ric-8A/NCS-1 R148A, R151A
Ric-8A/NCS-1 D37A,Y52A
Ric-8A/NCS-1 3M
Ric-8A/NCS-1 4M
Ric-8A L424A, M425A,/NCS-1 W30A
IP: anti-NCS-1
WB: anti V5
INPUT
Ric8a-V5
INPUT
NCS-1

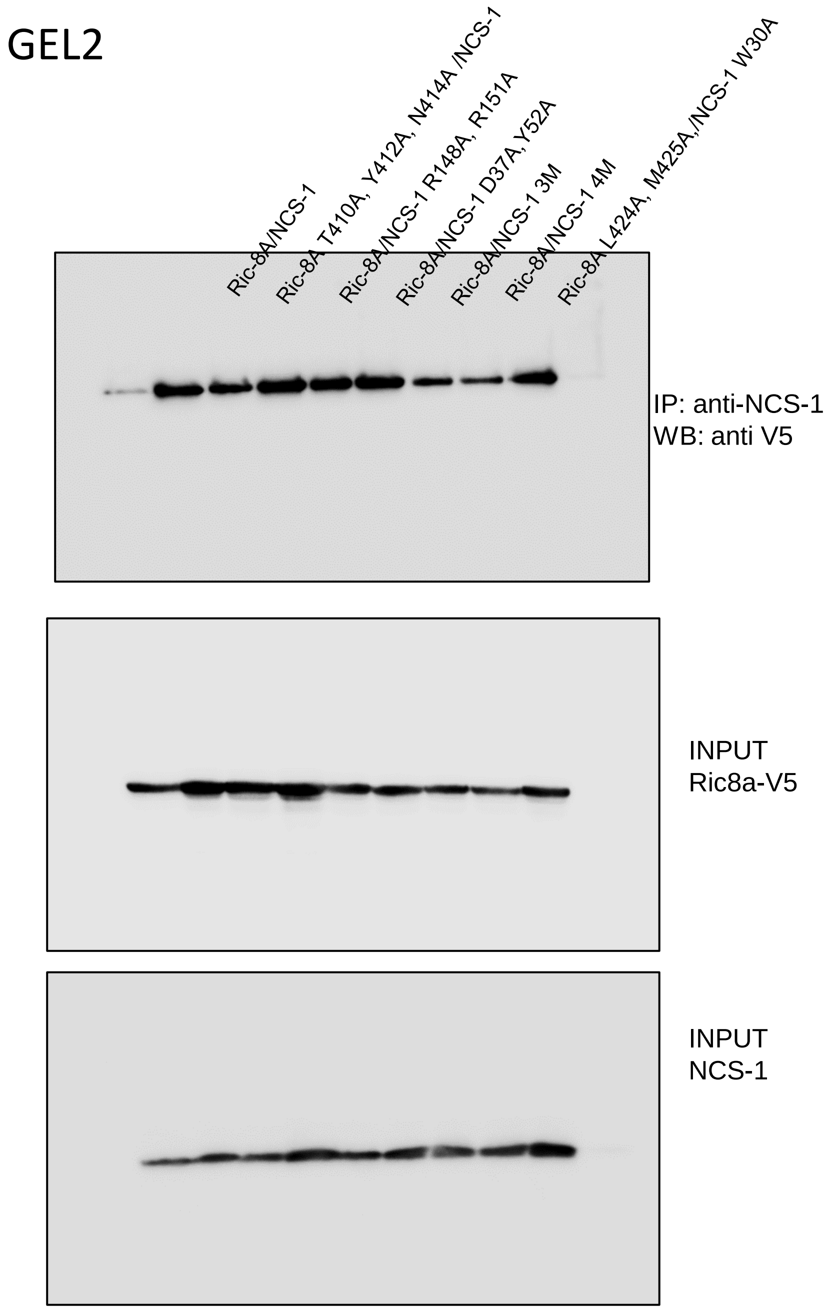
GEL2
Ric-8A/NCS-1
Ric-8A T410A, Y412A, N414A /NCS-1
Ric-8A/NCS-1 R148A, R151A
Ric-8A/NCS-1 D37A,Y52A
Ric-8A/NCS-1 3M
Ric-8A/NCS-1 4M
Ric-8A L424A, M425A,/NCS-1 W30A
IP: anti-NCS-1
WB: anti V5
INPUT
Ric8a-V5
INPUT
NCS-1

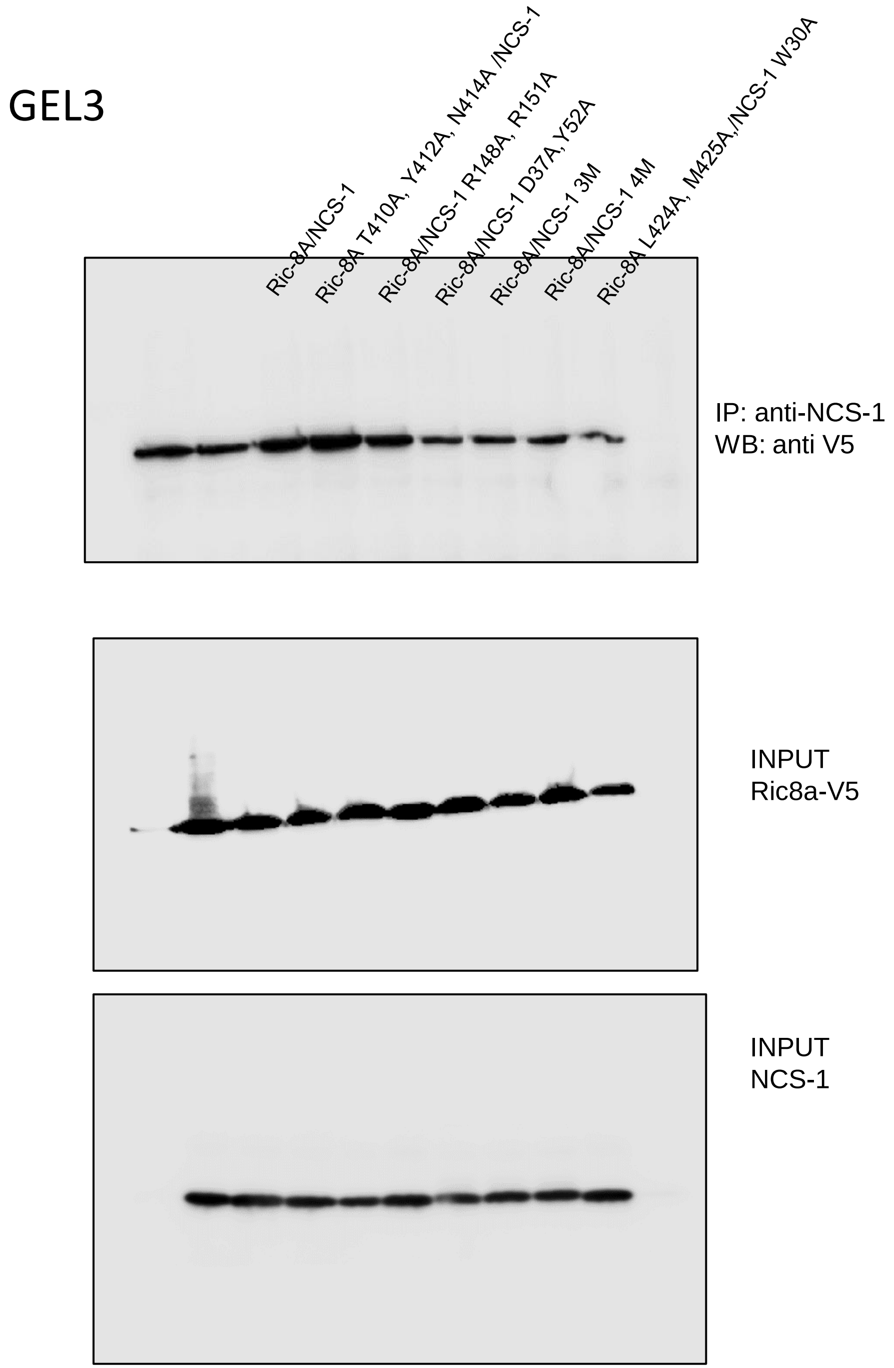
GEL3
Ric-8A/NCS-1
Ric-8A T410A, Y412A, N414A /NCS-1
Ric-8A/NCS-1 R148A, R151A
Ric-8A/NCS-1 D37A,Y52A
Ric-8A/NCS-1 3M
Ric-8A/NCS-1 4M
Ric-8A L424A, M425A,/NCS-1 W30A
IP: anti-NCS-1
WB: anti V5
INPUT
Ric8a-V5
INPUT
NCS-1
