# Supplementary material for: The neuronal calcium sensor NCS-1 regulates the phosphorylation state and activity of the Gα chaperone and GEF Ric-8A
Source: eLife. 2023 Nov 29;12:e86151. doi: 10.7554/eLife.86151 (PMC10732572; doi:10.7554/eLife.86151)
Supplement: Figure 5—source data 1. [file elife-86151-fig5-data1.zip › SourceData-Fig6/Figure 6A raw data legend.pdf]

A

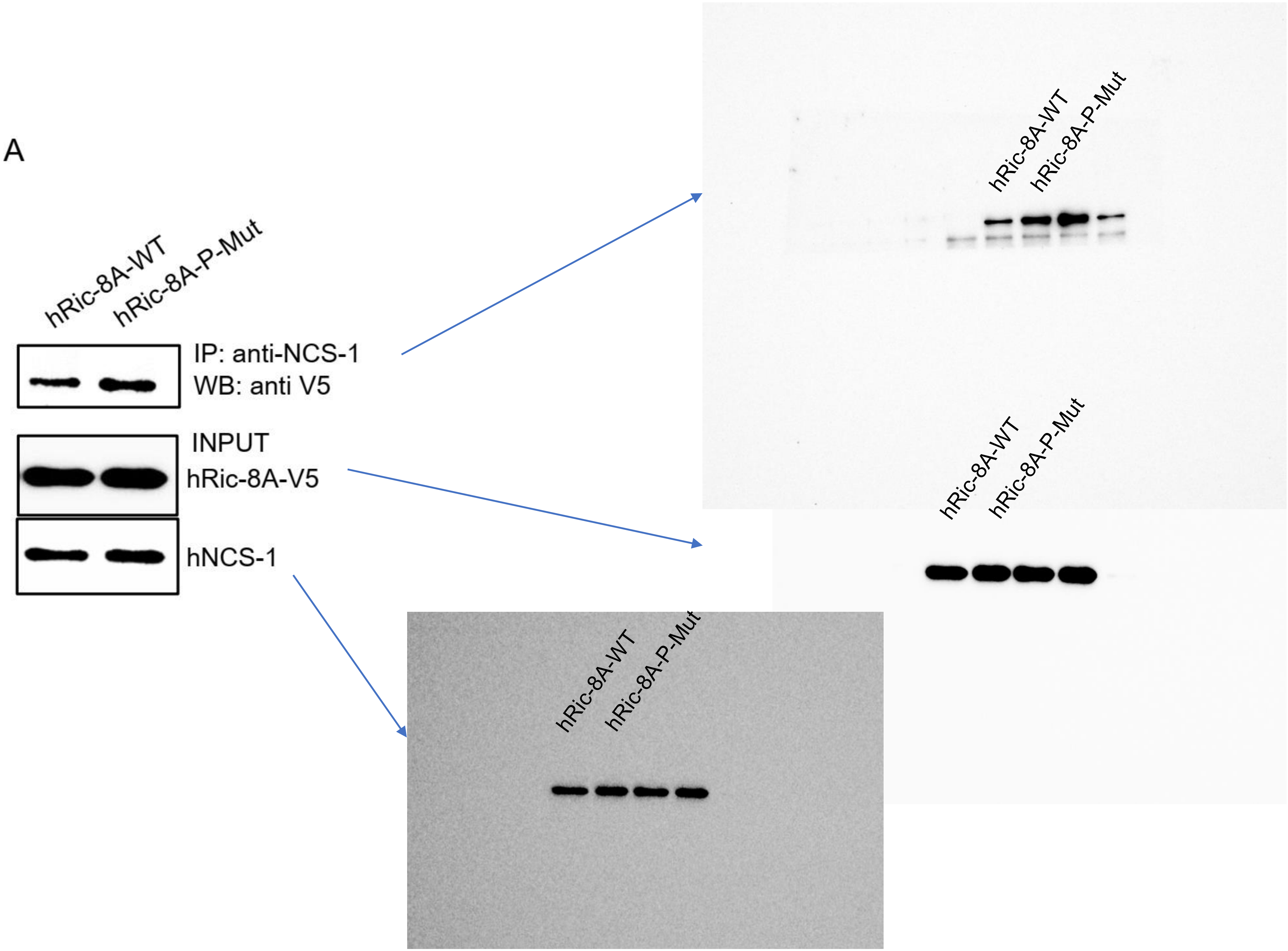
hRic-8A-WT
hRic-8A-P-Mut
IP: anti-NCS-1
WB: anti V5
INPUT
hRic-8A-V5
hNCS-1
hRic-8A-WT
hRic-8A-P-Mut
hRic-8A-WT
hRic-8A-P-Mut
hRic-8A-WT
hRic-8A-P-Mut
